# Predicting cervical cancer among women living with HIV/AIDS at public health facilities in a resource-limited setting in Ethiopia using machine learning analysis

**DOI:** 10.1007/s12672-026-05043-8

**Published:** 2026-04-24

**Authors:** Muluken Belachew Mengistie, Simane Kefale Mengstie, Gizaw Hailiye Teferi, Aynadis Worku Shume, Wubete Lule Ayalew, Mezigebu Lule Ayalew, Abraham Keffale Mengistu, Sefefe Birhanu Tizie, Ayenew Sisay Gebeyew, Misgie Kassanew Terefe, Lijalem Megibaru Enyew

**Affiliations:** 1 Department of Health Informatics, College of Medicine and Health Science, Debre Markos University, Debre Markos, Ethiopia; 2 Department of Midwifery, College of Medicine and Health Science, Debre Markos University, Debre Markos, Ethiopia; 3 Department of Gynecology and Obstetrics, School of Medicine, College of Medicine and Health Science, University of Gondar, Gondar, Ethiopia; 4 Zonal Health Department, East Gojjam Zone, Debre Markos, Ethiopia; 5 Department of Software Engineering, Institute of Technology, Debre Markos University, Debre Markos, Ethiopia

**Keywords:** Anti-retroviral therapy, Cervical cancer, Machine learning models, Predictors

## Abstract

**Introduction:**

Cervical cancer is a malignancy associated with human immunodeficiency virus, characterized by abnormal cervical cell mutations. Machine learning techniques offer valuable support for early detection and prediction of cervical cancer, potentially lowering screening and treatment costs. This study specifically targeted women living with human immunodeficiency virus, aiming to identify the most significant predictors of cervical cancer and to determine the most effective supervised machine learning model for its prediction within this population.

**Methods:**

This study employed a multi-center, cross-sectional design using a secondary dataset from the smart care systems of four antiretroviral therapy clinics in central Debre Markos town. To determine the most relevant predictors, seven machine learning models, Logistic Regression, Random Forest, K-Nearest Neighbors, Support Vector Machine, Decision Tree, Extreme Gradient Boosting, and AdaBoost were implemented to identify the top-performing model. Model performance was assessed using the confusion matrix and the Area under the Receiver Operating Characteristic Curve.

**Results:**

The findings indicated that adherence at enrollment, screening visit type, and nutritional status, months on anti-retroviral therapy, follow-up status, and weight were highly important predictors of cervical cancer. Among the evaluated models, the K-Nearest Neighbors model outperformed the others, achieving the highest accuracy of 98% and an Area under the Receiver Operating Characteristic Curve of 0.68.

**Conclusion:**

As demonstrated in this study, the K-Nearest Neighbors model showed the best performance in effectively predicting cervical cancer among women living with human immunodeficiency virus. Strengthening nutritional support interventions, improving follow-up mechanisms, and enhancing anti-retroviral therapy adherence counseling programs may collectively contribute to reducing the risk of cervical cancer among women living with human immunodeficiency virus. Future research should focus on validating the predictive model across diverse geographic regions and healthcare contexts to enhance its generalizability, robustness, and practical applicability.

## Introduction

Cervical cancer is a malignancy linked to human immunodeficiency virus acquired immunodeficiency syndrome (HIV/AIDS), characterized by abnormal changes in cervical cells that grow uncontrollably, forming tumors. These cancerous cells can invade nearby tissues and metastasize to other parts of the body if not treated promptly [1]. Globally, cervical cancer is the most common cancer among women, affecting around 24.6 million individuals, and ranks as the second most prevalent cancer in Africa, with a prevalence of 25.2%, highest in Eastern Sub-Saharan Africa [2].

The two main types of cervical cancer are squamous cell carcinoma and adenocarcinoma. Women over 30 years old are at greater risk, and more than 70% of cases are caused by human papillomavirus (HPV), which is preventable and treatable if detected early [3]. In Ethiopia, over 33.7 million women aged 15 and above are at risk. Regular screening through visual inspection with acetic acid (VIA) or Pap tests is the most effective approach for early detection, improving treatment outcomes and reducing the risk of invasive cancer and mortality [4].

To combat cervical cancer, the World Health Organization (WHO) introduced the 90-70-90 strategy in 2020, aiming to eliminate cervical cancer by 2030. This strategy targets 90% HPV vaccination coverage, screening 70% of women with high-performance tests at least twice, and ensuring treatment for 90% of diagnosed cases. Similarly, Ethiopia’s National Cancer Control Plan (2016–2020) aimed for at least 80% screening coverage using VIA and offered free services in all government health facilities [5].

Although cancer treatment has evolved, early detection remains critical, as cervical cancer is often asymptomatic in its initial stages. Screening and testing are essential for identifying precancerous changes, yet the lack of effective screening, HPV vaccination, and early detection programs results in most cases and deaths occurring in low- and middle-income countries [6, 7]. Once diagnosed, cervical cancer can have severe physical, psychological, and financial impacts, including fatigue, pain, heavy bleeding, reduced sexual activity, anxiety, depression, and reduced life expectancy [8].

Cervical cancer is largely preventable through vaccination, screening, and timely treatment. Worldwide, it is the third most common cancer among women, with 604,000 new cases annually, causing approximately 342,000 deaths each year, 90% of which occur in low- and middle-income countries. By 2030, deaths are projected to rise to 443,000, a 67% increase [2, 9]. In Africa, around 119,284 new cases are diagnosed yearly, leading to 81,687 deaths among women aged 14–44. Sub-Saharan Africa has an incidence rate of 25.2%, a mortality rate of 23.2%, and a prevalence of 27.6%, with notable variations between countries. Malawi records the highest incidence and mortality globally, while Uganda has the second-highest incidence in East Africa [8–10].

In Ethiopia, about 7,000 new cases and 5,000 deaths occur annually, with incidence, mortality, and prevalence rates of 17.3%, 16.5%, and 18.2%, respectively [7]. The high prevalence is driven by a fragile healthcare system and high HIV/AIDS rates. Women living with HIV are six times more likely to develop cervical lesions, making Ethiopia’s 534,000 HIV-positive women especially vulnerable [11]. Key risk factors for cervical cancer include early sexual activity, age over 35, multiple sexual partners, history of sexually transmitted infections, long-term contraceptive use, multiple pregnancies, young age at childbirth, low education and income, advanced HIV, high viral load, low cluster of differentiation 4 (CD4) count, poor condom use, lack of vaccination, and certain marital statuses [12–14].

Despite preventive measures such as screening and HPV vaccination, cervical cancer remains a major public health concern, making early detection essential for reducing mortality. Accurately predicting cervical cancer in HIV-positive women on antiretroviral therapy (ART) is particularly challenging, as the absence of reliable predictive tools limits the identification of high-risk individuals, resulting in missed opportunities for timely intervention and preventable adverse outcomes [15]. Advances in artificial intelligence, especially machine learning, offer promising solutions by enabling more precise and efficient disease prediction, identifying high-risk patients, and supporting timely clinical decision-making [16–20]. These models are particularly valuable in resource-limited settings, where they facilitate personalized care, closer patient monitoring, targeted treatment adjustments, and optimized allocation of healthcare resources. Additionally, they can anticipate comorbidities such as tuberculosis, cardiovascular diseases, and opportunistic infections, thereby improving overall outcomes for HIV-positive women [21–24].

Previous studies on cervical cancer have overlooked HIV-positive women, relied on small samples, and lacked advanced analytical methods. This study addresses these gaps by developing a machine-learning model to predict cervical cancer among HIV-positive women on ART [10, 25]. The findings of this study are expected to inform public health strategies, strengthen cervical cancer screening programs, optimize ART and preventive interventions, and guide policymakers in resource-limited settings. Ultimately, integrating machine learning predictive tools into healthcare systems can enhance individualized patient care, reduce cervical cancer incidence and mortality, and improve overall health system efficiency [26, 27].

## Methods

### Study area and period

A retrospective cross-sectional study was conducted in Debre Markos Town, located in the East Gojjam Zone of Northwest Ethiopia. Debre Markos has a total population of 262,497, comprising 129,921 males and 132,576 females. Data for this study were extracted between March and April 2025. To mitigate the burden of cervical cancer, the Debre Markos city administration has independently implemented a range of preventive and control measures. These include community-based health education, cervical cancer screening programs, and treatment services for diagnosed cases.

### Dataset description

This study utilized secondary data from 2,337 records collected at four ART clinics in Debre Markos town between December 1, 2020 and December 1, 2024. The data were extracted from the clinics’ Smart Care system and include information on HIV-positive women since the initiation of ART. A retrospective chart review was conducted to collect relevant clinical and demographic data used to develop and evaluate a machine learning model for predicting cervical cancer in this population. Women living with HIV/AIDS aged 15–49 years who were enrolled in ART follow-up care were included, in accordance with standard treatment protocols. A minimum ART duration of six months was required to ensure the availability of adequate treatment response data. Women who were eligible for cervical cancer screening but had not been screened, as well as those with missing critical medical records (e.g., follow-up status), were excluded from the analysis.

### Data collection tool and procedures

In this study, data collection from the smart care system was done in Excel format to retrieve relevant patient information from the electronic medical record ART database from different ART facilities. The extraction form captured essential demographic details, including age and sex. These factors were considered crucial for understanding the social context of the patients and assessing potential cancer risk contributors. Data quality control measures were implemented throughout the study to guarantee reliability and validity. This included performing consistency checks and addressing any missing entries. Experienced clerks supervised data entry, using predefined validation rules to flag any inconsistencies.

### Data preprocessing

After data acquisition, the dataset underwent an initial data-cleaning phase. Missing numerical values were handled using mean imputation, while missing categorical values were addressed using mode imputation. Categorical variables were then encoded using one-hot encoding, with each category represented as a binary indicator (0 or 1). To address class imbalance, both Synthetic Minority Oversampling Technique (SMOTE) and Adaptive Synthetic Sampling (ADASYN) were evaluated. Although both techniques demonstrated comparable predictive performance, SMOTE was selected due to its methodological simplicity, computational efficiency, and greater model stability. Unlike ADASYN, which emphasizes difficult-to-learn minority instances and may amplify noise, SMOTE generates synthetic samples more uniformly within minority class neighborhoods, resulting in more robust and generalizable decision boundaries. Given the absence of a significant performance advantage with ADASYN, SMOTE was retained as the preferred imbalance-handling method. To tackle multicollinearity and enhance predictive accuracy, correlation heat maps were utilized as needed. Feature importance was subsequently assessed using the Random Forest (RF) algorithm, which ranks relevant predictors, including age, marital status, occupation, educational status, residence, weight, religion, alcohol use, smoking, khat chewing, early age of the first sex, age of pregnancy, abortion, parity, condom use, sexually transmitted infection (STI), vaginal wall abnormality, multiple sexual partners, CD4 count, viral loads, WHO HIV staging, tuberculosis preventive therapy, and months on ART based on their contribution to model performance. Following preprocessing and feature selection, the dataset was split into training and testing sets, with 80% of the data used for model training and 20% reserved for evaluating performance on previously unseen data. This study used Python (version 3.11) for data management and analysis, primarily employing the Pandas library.

### Model training

The training process began with RF feature selection to identify the most relevant predictors of cervical cancer in women living with HIV. Seven classifiers were then trained: Logistic Regression (LR), Decision Tree (DT), Random Forest (RF), K-Nearest Neighbors (KNN), Support Vector Machine (SVM), Extreme Gradient Boosting (XGB), and AdaBoost (ADB). These algorithms were chosen based on prior studies demonstrating their effectiveness in healthcare-related machine learning, including cervical cancer prediction [18, 28].

### Model evaluation

After training, the models were systematically evaluated to determine the most effective classifier for predicting cervical cancer in women living with HIV. Performance metrics included accuracy, precision, recall, F1-score, and the area under the ROC curve (AUC-ROC) [29].

## Results

### Socio-demographic and reproductive health characteristics

The study included 2,337 participants aged 15 to 49 years. Among them, the majority (86.65%) were over 30 years old, and most (57.2%) had a body weight of 51–65 kg. Additionally, 63.63% of the participants were undergoing cervical cancer screening for the first time, with all tested using the VIA method. Furthermore, 92.5% reported using family planning methods, and nearly all (99.1%) received negative results for cervical cancer screening (Table 1).

**Table 1:** Socio-demographic characteristics of the study participants in Debre Markos town

Feature	Category	Frequency (*n*)	Percent (%)
Age	15–30	312	13.4
	31–49	2025	86.6
Weight	38–50	672	28.8
	51–65	1336	57.2
	> 65	329	14.1
Screening Visit Type	1st time	1481	63.4
	Re-screening	827	35.4
	Post treatment	29	1.2
Screening Accepted	Yes	2317	99.1
	No	20	0.9
Screening Mechanism	VIA	2336	99.9
	HPV	1	0.1
Family Planning	Yes	2162	92.5
	No	175	7.5
Screening result	Negative	2315	99.1
	Positive	22	0.9

### Clinical characteristics of respondents

The majority of participants (96.5%) showed good adherence to ART, indicating generally positive treatment behavior. Furthermore, most participants (99.8%) fell within the range of Body Mass Index (BMI) 18–25. Additionally, 84.5% of participants had been on ART for more than 100 months, a duration associated with satisfactory immune function, life span, and viral suppression. Poor adherence over time, however, may make managing the viral infection more challenging and lead to drug resistance (Table 2).

**Table 2:** Clinical characteristics of study participants in Debre Markos Town

Features	Category	Frequency (*n*)	Percent (%)
ART Follow-up status	Alive	2330	99.7
	Restart	7	0.3
ART Regimen at enrollment	1a	40	1.7
	1b	39	1.7
	1c	2182	93.4
	1d	19	0.8
	1e	19	0.8
	1f	38	1.6
Adherence at enrollment	Good	2255	96.5
	Fair	72	3.1
	Poor	10	0.4
Months on ART	3-100 months	1974	84.5
	101–200 months	339	14.5
	> 200 months	24	1.0
Nutritional Status	BMI < 18	2	0.1
	BMI18-25	2333	99.8
	BMI > 25	2	0.1

### Balancing dataset

**Fig. 1:**
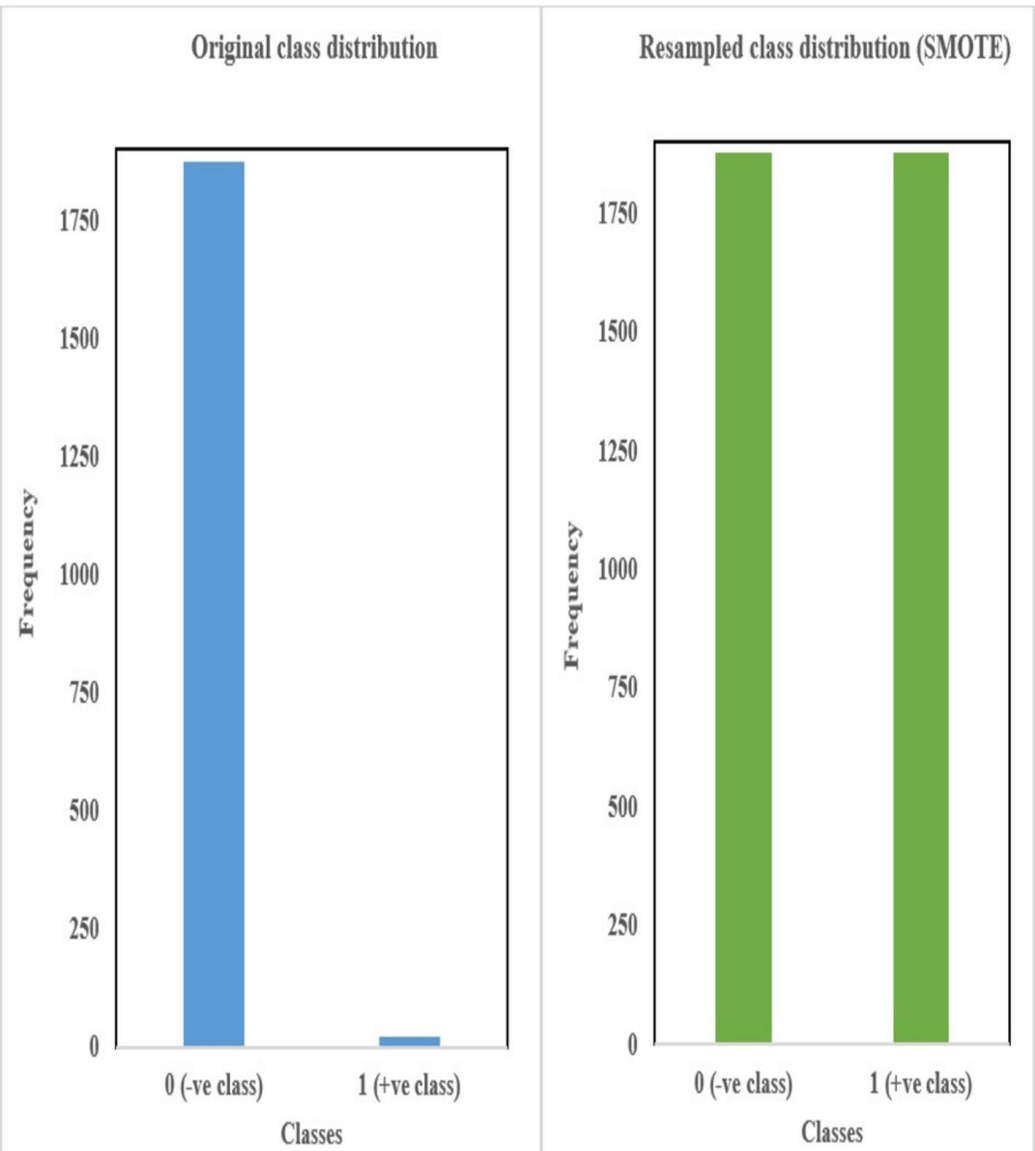
Class distribution before smote and after smote

Descriptive statistics showed that only 0.9% of women living with HIV in the dataset were diagnosed with cervical cancer, reflecting a pronounced class imbalance, with roughly 80% of cases belonging to the negative class. To address this, the SMOTE oversampling technique generated 1,853 synthetic samples for the minority (positive) class, creating a balanced dataset of 1,875 instances per class. This balanced dataset was then used to train the machine learning models with an 80:20 train-test split (Fig. 1).

### Model evaluation

The evaluation results showed that the KNN model outperformed all other classifiers, achieving 98% accuracy, precision, recall, and F1 Score in predicting cervical cancer, making it the most effective model in this study. Random Forest and XGB also performed well, each with 93% accuracy, while DT, LR, ADB, and SVM showed lower performance at 90% accuracy (Table 3).

**Table 3:** Model Evaluation metrics and Auroc for this study

Algorithm		Accuracy (%)	Precision (%)	Recall (%)	F1_score (%)	AUROC
XGB	Unbalanced	0.99	0.99	1.00	1.00	0.94
	Balanced	0.93	0.98	0.93	0.95	0.78
LR	Unbalanced	0.99	0.99	1.00	0.99	0.92
	Balanced	0.90	0.98	0.90	0.94	0.65
RF	Unbalanced	0.99	0.99	1.00	0.99	0.94
	Balanced	0.93	0.98	0.93	0.95	0.79
DT	Unbalanced	0.99	0.99	1.00	0.99	0.88
	Balanced	0.92	0.98	0.92	0.95	0.79
SVM	Unbalanced	0.99	0.99	1.00	0.99	0.92
	Balanced	0.92	0.98	0.92	0.95	0.72
KNN	Unbalanced	0.99	0.99	1.00	0.99	0.88
	Balanced	0.98	0.98	0.98	0.98	0.68
ADB	Unbalanced	0.99	0.99	1.00	0.99	0.93
	Balanced	0.90	0.98	0.90	0.93	0.81

### Model performance comparison

**Fig. 2:**
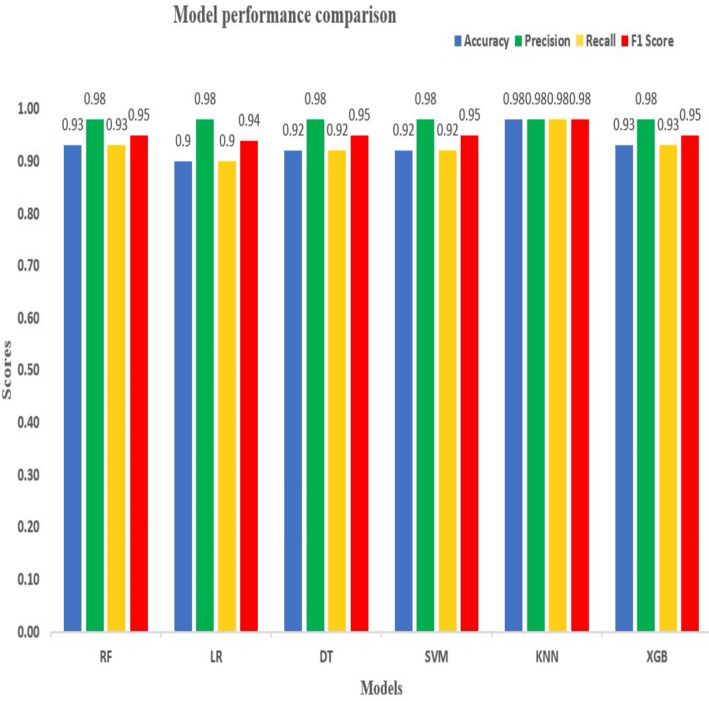
Model performance comparison using different metrics

The model comparison plot shows that KNN outperformed all other algorithms, achieving 98% across accuracy, precision, recall, and F1-score. RF and XGB performed moderately well, while SVM, AdaBoost, DT, and LR showed lower performance (Fig. 2).

**Fig. 3:**
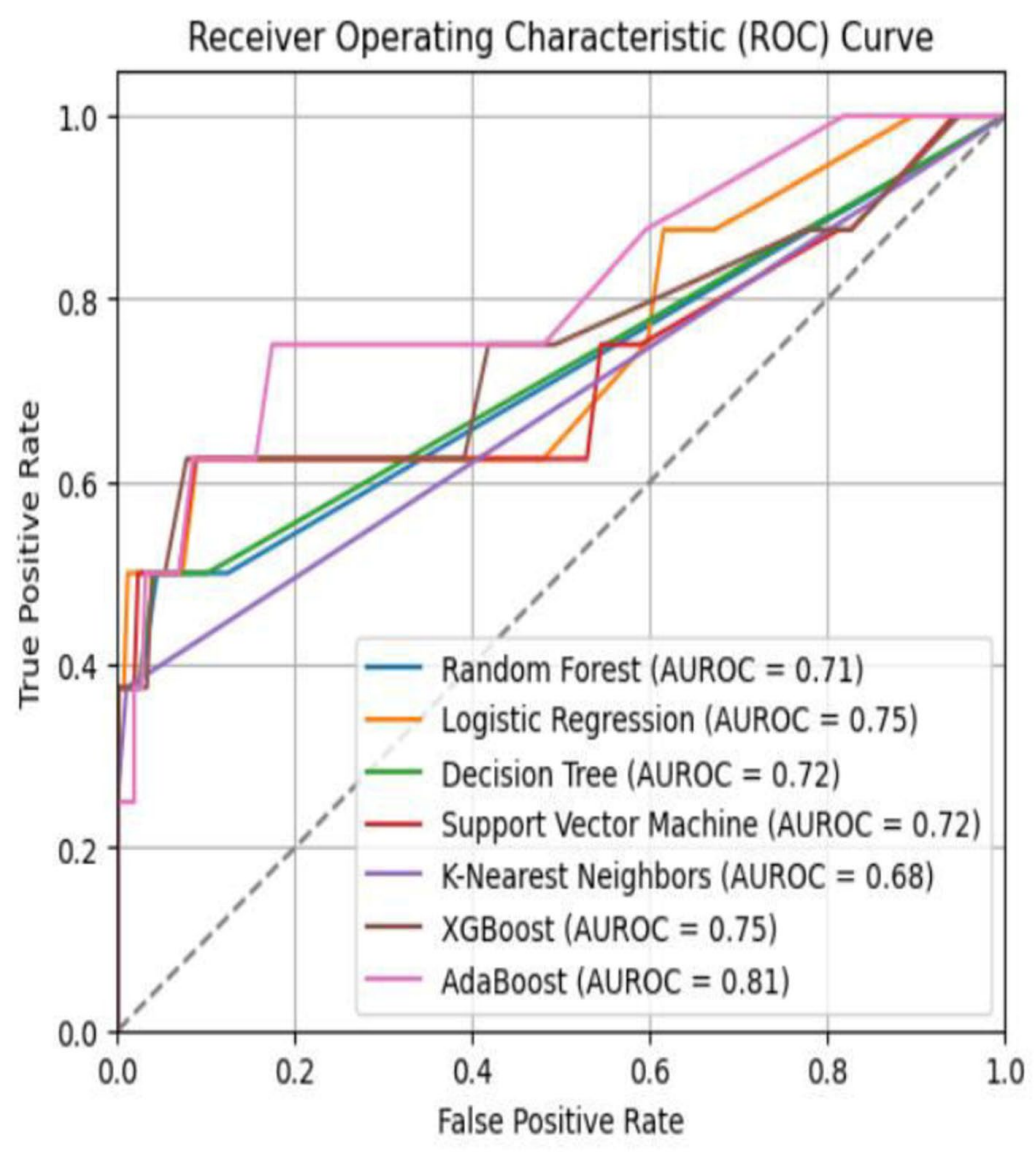
ROC curve for machine learning models in predicting cervical cancer after smote

The ROC curve analysis illustrates model performance before and after dataset balancing. Initially, all models showed perfect AUC scores of 1.00. After applying SMOTE, model performance became more realistic and generalizable. AdaBoost’s AUC dropped to 0.81, DT and SVM reached 0.72, while KNN scored 0.68. RF and XGB achieved AUCs of 0.71 and 0.75, respectively (Fig. 3).

**Fig. 4:**
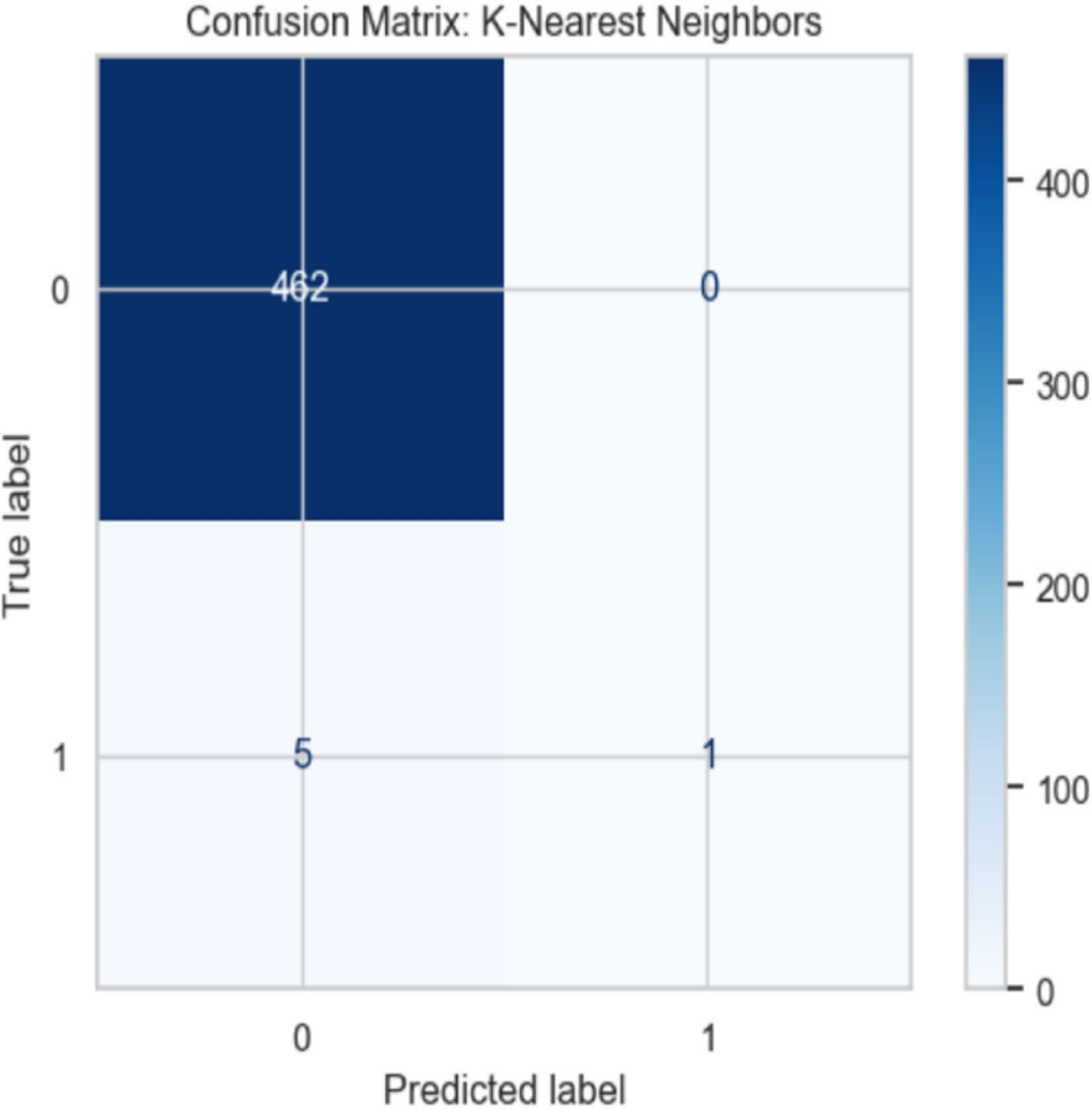
Confusion matrix of the KNN classifier cervical cancer prediction among women with HIV

A confusion matrix is a powerful tool for assessing a model’s performance by comparing its predicted outputs with the actual labels. It offers valuable insights into how effectively the model distinguishes between negative (0) and positive classes (1) and shows a confusion matrix of KNN (Fig. 4).

The KNN model demonstrated excellent overall performance across all evaluation metrics. It achieved an accuracy of 98%, indicating that the vast majority of predictions, both cervical cancer and non-cancer cases, were correctly classified. The precision of 98% shows that when the model predicts cervical cancer, it is correct 98% of the time, reflecting a very low false-positive rate. Similarly, the recall of 98% indicates that the model successfully identifies 98% of all true cervical cancer cases, demonstrating a very low false-negative rate. The F1-score of 98% further confirms a strong balance between precision and recall, highlighting the model’s robustness and reliability in effectively detecting cervical cancer while minimizing misclassification.

### Feature importance analysis using K-Nearest neighbors

**Fig. 5:**
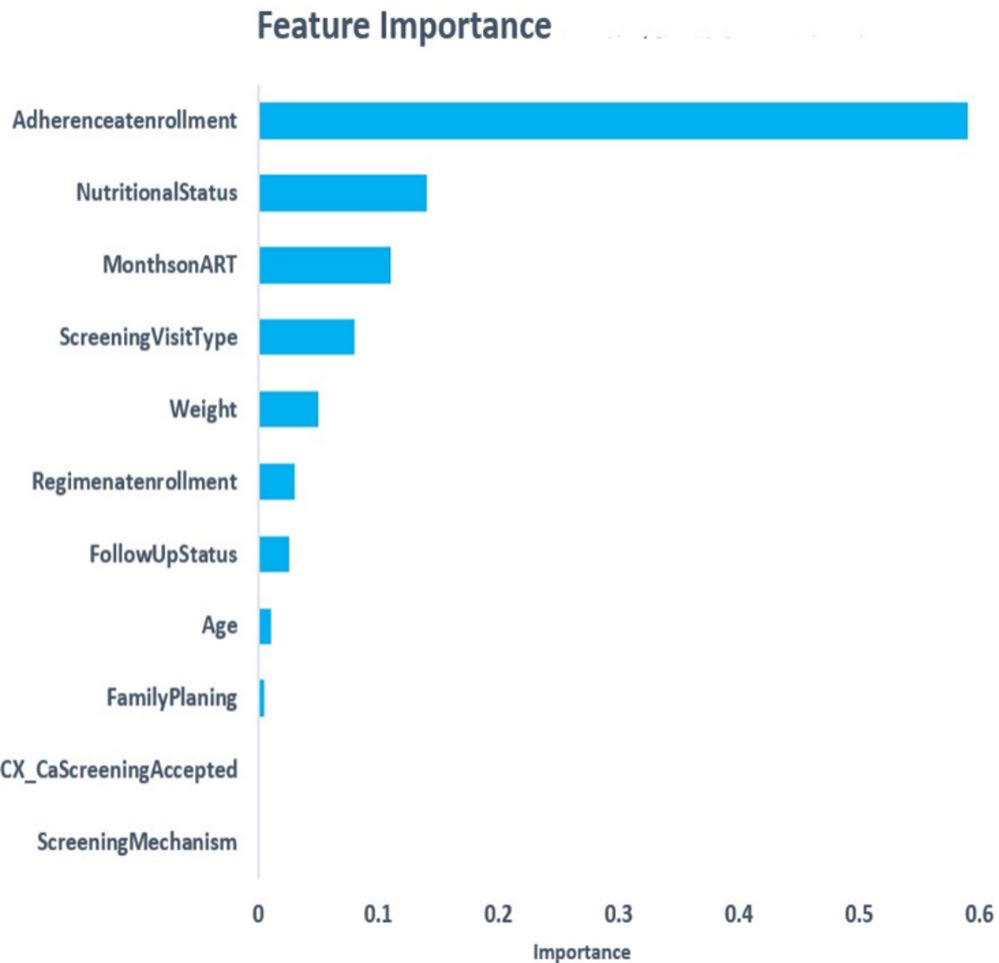
Feature importance ranking using K-Nearest neighbors

This study utilized the KNN technique to determine the most significant predictors of cervical cancer in women living with HIV. The KNN model assessed the importance of each predictor in relation to the screening outcome. By calculating feature importance scores throughout the dataset, the study identified and ranked the top predictors in descending order according to their impact on the outcome. Notable variables included adherence at enrollment, nutritional status, months on ART, screening visit type, weight, regimen at enrollment, and follow-up status (Fig. 5).

## Discussion

The model comparison plot in this study showed that KNN outperformed all other algorithms, achieving 98% accuracy, precision, recall, and F1-score, highlighting its strong ability to predict cervical cancer. RF and XGB showed moderate performance, whereas SVM, ADB, DT, and LR performed less effectively. KNN’s strength lies in its simplicity, flexibility, and non-parametric nature, making it well-suited for complex, non-linear data. Comparable studies reported lower performance. For instance, a study conducted in Uganda indicates that RF achieved 90% accuracy and 90.1% AUROC [30]. Similarly, another study done in Iran also shows that DT reached 95.55% accuracy, 90.48% precision, 100% recall, and 95.25% AUC [14]. Moreover, a study conducted in Bangladesh reports that DT and KNN models attained 96.41% accuracy [31]. Conversely, studies in Saudi Arabia reported that 100% accuracy using RF, DT, and ADB [18], and in the United States, XGB achieved 96.5% accuracy [32]. The differences observed between this study and others may be due to variations in participants’ demographics, including age, ethnicity, and health status. Dataset size also plays a key role, as smaller datasets can increase the risk of overfitting, whereas larger datasets enable more robust model training. Although the KNN model achieved high accuracy, the AUC values were relatively low, likely due to overlapping feature distributions and algorithm sensitivity to data characteristics. Preprocessing steps, including feature selection and class balancing, were applied, but residual limitations may have constrained discriminative performance [33, 34].

This study identified the most important predictors using KNN feature importance, including adherence at enrollment, months on ART, regimen at enrollment, nutritional status, and screening visit type. The top predictor was ART adherence, a critical factor influencing cancer risk. Early initiation of ART supports sustained viral suppression and immune recovery, thereby lowering the risk of opportunistic infections and related cancers [35]. Highlighting ART adherence as a key predictor underscores the importance of timely ART initiation and continuous adherence support programs in cancer prevention.

Duration of ART was also identified as a significant predictor of cervical cancer. Prolonged ART use often results in undetectable viral loads, reducing chronic immune activation and inflammation. Maintaining viral suppression is crucial for preventing disease progression and complications from co-infections such as HPV [36]. Longer ART exposure enhances the immune system’s ability to clear HPV infections, lowering the risk of Cervical Intraepithelial Neoplasia (CIN) and invasive cervical cancer. Nutritional status, another key predictor identified by KNN, also plays a critical role in cancer risk among women living with HIV. Proper nutrition reduces the risk of cervical cancer, aids disease management, and affects both CIN progression and HPV persistence [37].

The type of screening visit was also identified as an important predictor, as regular cervical cancer screening is vital for early detection, prevention, and effective management. This aligns with Ethiopian research showing that early detection and treatment can prevent up to 80% of cervical cancer cases [25]. Screening enhances vaccination efforts, is cost-effective, and significantly benefits public health, particularly in resource-limited settings. Similarly, the ART regimen plays a key role in reducing cervical cancer risk among women living with HIV by restoring immune function and limiting HPV progression. Studies indicate that women on ART have a lower risk of invasive cervical cancer compared to those not receiving treatment [38], highlighting the importance of early ART initiation and appropriate regimen selection.

Factors such as age, family planning, and screening practices contribute to the multifactorial risk of HIV-related cervical cancer. Risk increases with age, particularly in women aged 30 and older, who are more prone to persistent HPV infections. Long-term use of oral contraceptives has been associated with a slightly elevated risk, which decreases after discontinuation. Regular, cost-effective cervical cancer screening is essential for early detection of precancerous changes, enabling timely intervention to prevent cancer progression [39–41].

## Conclusion

Early detection significantly improves the effectiveness of screening and treatment in both the pre-cancerous and cancer stages. Being aware of the signs and symptoms of cervical cancer can help reduce delays in diagnosis. This study found several key predictors of cervical cancer among women living with HIV, including adherence at enrollment, nutritional status, months on ART, the type of screening visit, and the regimen at enrollment. This study showed that the KNN model predicts cervical cancer effectively in women living with HIV. However, the generalizability of these models is limited, as they were developed using data exclusively from health facilities in Debre Markos city and may not perform comparably in settings with different health system characteristics, sociodemographic conditions, cervical cancer prevalence rates, testing protocols, or healthcare infrastructure. Future research should prioritize prospective validation and evaluation across diverse regions and healthcare settings to enhance generalizability and support practical implementation. Additionally, exploring further predictive features or alternative modeling approaches may help improve AUC and overall discriminative performance.

## Data Availability

All relevant data are available within the paper and from the corresponding author.

## Abbreviations

ADB: AdaBoost
ADASYN: Adaptive synthetic sampling
AIDS: Acquired immune deficiency syndrome
BMI: Body mass index
CD4: Cluster of differentiation 4
CIN: Cervical intraepithelial neoplasia
HIV: Human immune virus
HPV: Human papillomavirus
KNN: K-Nearest neighbor
LR: Logistic regression
RF: Random forest
SMOTE: Synthetic minority oversampling technique
STI: Sexually transmitted infection
SVM: Support vector machine
VIA: Visual inspection acetic acid
WHO: World Health Organization
XGB: Extreme gradient boosting
